# Ginsenoside Rb1 Protects Rat Neural Progenitor Cells against Oxidative Injury

**DOI:** 10.3390/molecules19033012

**Published:** 2014-03-07

**Authors:** Na Ni, Qiang Liu, Huixia Ren, Di Wu, Chuanming Luo, Peng Li, Jian-Bo Wan, Huanxing Su

**Affiliations:** State Key Laboratory of Quality Research in Chinese Medicine, Institute of Chinese Medical Sciences, University of Macau, Macao 999078, China; E-Mails: lovemonicamacau@gmail.com (N.N.); liuqiangsh@126.com (Q.L.); yb27526@umac.mo (H.R.); dierwu@gmail.com (D.W.); 15989166366@163.com (C.L.); pengli@umac.mo (P.L.); JBWan@umac.mo (J.-B.W.)

**Keywords:** ginsenoside Rb1, neural progenitor cells, oxidative stress, Nrf2

## Abstract

Ginseng, the root of *Panax ginseng* C.A. Meyer, has been used as a tonic to enhance bodily functions against various ailments for hundreds of years in Far Eastern countries without apparent adverse effects. Ginsenoside Rb1, one of the most active ingredients of ginseng, has been shown to possess various pharmacological activities. Here we report that Rb1 exhibits potent neuroprotective effects against oxidative injury induced by *tert*-butylhydroperoxide (*t*-BHP). Lactate dehydrogenase (LDH) assay demonstrated that incubation with 300 µm *t*-BHP for 2.5 h led to a significant cell loss of cultured rat embryonic cortex-derived neural progenitor cells (NPCs) and the cell viability was pronouncedly increased by 24 h pretreatment of 10 µm Rb1. TUNEL staining further confirmed that pretreatment of Rb1 significantly reduced the cell apoptosis in *t*-BHP-induced oxidative injury. Real time PCR revealed that pretreatment with Rb1 activated Nrf2 pathway in cultured NPCs and led to an elevated expression of HO-1. The results of the present study demonstrate that Rb1 shows a potent anti-oxidative effect on cultured NPCs by activating Nrf2 pathway.

## 1. Introduction

In a biological system oxidative stress represents an imbalance between the production of reactive oxygen species (ROS) and the ability to repair damaged tissues. Oxidative stress increases the oxidation of varioius macromolecules, including lipid, protein, DNA and RNA, causing neuronal dysfunction and/or death [1,2]. Oxidative stress has emerged as a causative factor in the pathogenesis and progression of neurodegenerative disorders such as Alzheimer’s disease, Parkinson’s disease, amyotrophic lateral sclerosis and Huntington’s disease [3]. Hence, studies focusing on finding potent antioxidants are of vital significance and can form the basis of new therapies targeting the treatment of these neurodegenerative disorders.

Ginseng, the root of *Panax ginseng* C.A. Meyer, has been used extensively in China for over 2,000 years to treat a series of diseases. Ginsenoside Rb1, a pharmacologically active compound of ginseng, has received a lot of attention due to its biological properties. Many studies provide evidence that ginsenoside Rb1 possesses potent neuroprotective effects on cortical neurons and dopaminergic neurons against glutamate toxicity [4,5], protects against cerebral ischemia in rats by promoting neurogenesis [6], enhances nerve growth factor (NGF)-mediated neurite outgrowth of cultured chick embryonic dorsal root ganglia [7], prevents MPP^+^-induced apoptosis in PC12 cells [8], inhibits neuroinflammations in a rat model of Alzheimer’s disease [9], and improves spatial learning and increases hippocampal synaptophysin level in mice [10]. All these studies suggest that ginsenoside Rb1 has therapeutical potential in treatment of neurological disorders.

In the present study, we investigated the anti-oxidative effects of Rb1 on cultured neural progenitor cells (NPCs). NPCs are multipotent with a broad self-renewing potential and with the capacity to generate neurons, astrocytes and oligodendrocytes and have been demonstrated to be widely distributed in the embryonic CNS and also in the adult brain, where they are restricted to two main regions: the hippocampal dentate gyrus and the subventricular zones of the lateral ventricles [11,12]. The inherent biological properties of NPCs provide multiple strategies to treat various neurological dysfunctions. The results of the present study would provide evidence on whether ginsenoside Rb1 can protect NPCs against oxidative injury.

## 2. Results and Discussion

### 2.1. *In Vitro* Characterization of NPCs

**Figure 1:**
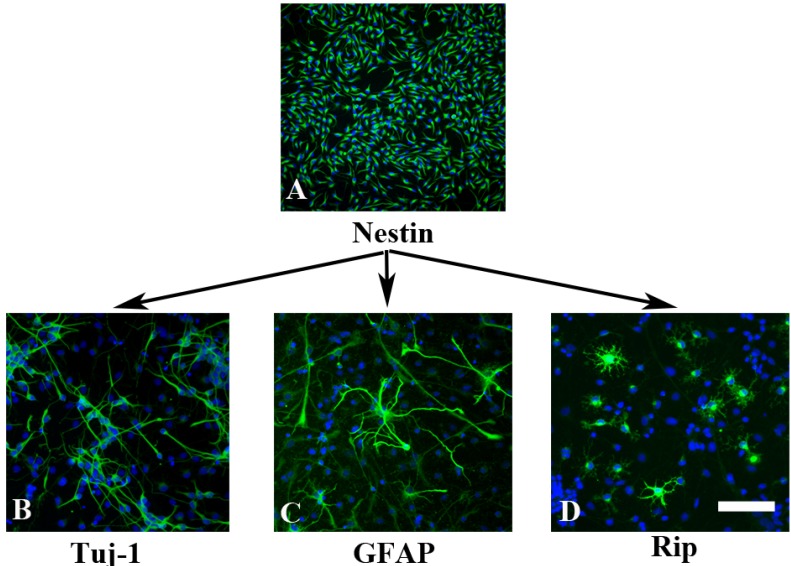
Characterization on NPCs. (**A**) NPCs were isolated from E-14.5 rat cortex and cultured in bFGF- and EGF-supplemented culture medium. Cells were either bipolar or multipolar with immunoreactive for nestin; (**B**–**D**) When bFGF and EGF were removed from the culture and replaced with 1% FBS for 5 days, NPCs successfully differentiated into βIII-tublin-positive neurons (**B**); GFAP-positive astrocytes (**C**); and Rip-positive oligodendrocytes (**D**). Scale bar: 150 µm in A; 50 µm in B–D.

With bFGF- and EGF-supplemented culture medium, the majority cells showed bipolar or multipolar morphology with small cell bodies and were immunoreactive for nestin, a marker for NPCs (Figure 1A), confirming that the cells remained in an immature stage. When bFGF and EGF was replaced with 1% FBS, NPCs began to differentiate. At the 5th day culture with this differentiating medium, NPCs successfully differentiated into βIII-tublin-positive neurons (Figure 1B), GFAP-positive astrocytes (Figure 1C) and Rip-positive oligodendrocytes (Figure 1D). There was still a very small population of cells with the morphology of undifferentiated cells that were positive for nestin immunoreactivity (data not shown).

### 2.2. Rb1 Pretreatment Reduced Oxidative Stress on Culture NPCs

**Figure 2:**
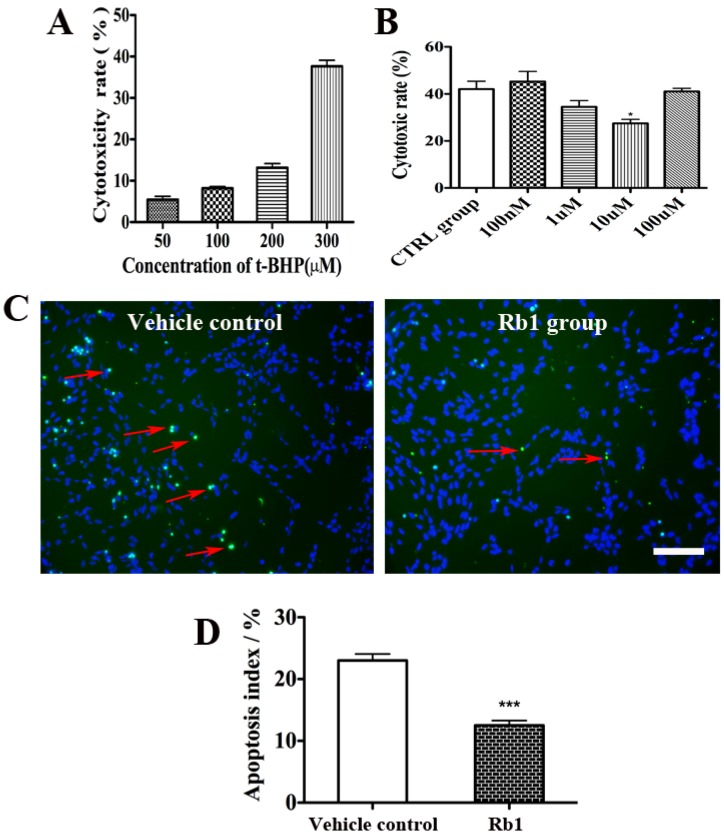
Rb1 pretreatment reduced oxidative stress on culture NPCs. (**A**) LDH assays revealed that *t*-BHP treatment has caused cell toxicity in a concentration-dependent manner; (**B**) LDH assays revealed that the pretreatment of 10 µM Rb1 for 24 h significantly attenuated *t*-BHP-induced cell injury compared with vehicle controls. Results are expressed mean ± SEM, *n =* 4, * *p <* 0.05 *versus* control group; (**C**) Representative photomicrographs of the TUNEL assay. The red arrow indicates apoptotic cells; (**D**) The percentage of TUNEL-positive cells was significantly reduced in Rb1-treated group compared with that in the vehicle-treated group. Eight representative fields per well and three wells per experiment were counted. Data were presented as means±SEM from six experiments. * *p <* 0.01 compared with the control group. Scale bar: 75 µm.

*tert*-Butylhydroperoxide (*t*-BHP) is used in a variety of oxidation processes like hydrogen peroxide (H_2_O_2_). It has a better stability as compared to H_2_O_2_. Therefore the present study employed *t*-BHP to establish the oxidative injury model. As shown in Figure 2A, *t*-BHP treatment induced cell toxicity in a concentration-dependent manner. NPCs treated with 50, 100, 200 and 300 µM *t*-BHP for 2.5 h led to a cytotoxicity rate 5.4% ± 0.47%, 8.2% ± 0.24%, 13.13% ± 0.6%, and 37.67% ± 0.84% respectively. Since a 35%–45% toxicity rate is considerated to be an optimal oxidative stress model, we investigated the anti-oxidative effect of Rb1 under conditions of oxidative injury induced by 300 µM *t*-BHP for 2.5 h.

We then investigated whether Rb1 pretreatment could reduce NPC death under oxidative injury conditions. After incubation with 300 µM *t*-BHP for 2.5 h, a LDH assay revealed that the pretreatment of 10 µM Rb1 for 24 h significantly attenuated *t*-BHP-induced cell injury compared with vehicle controls (Figure 2B). This cytoprotective effect was supported by TUNEL staining, which showed a significant reduced TUNEL positivity in Rb1-treated group compared with that in the vehicle-treated group (23% ± 0.38% in control group *vs.* 12.5% ± 0.28% in Rb1 group, *p <* 0.001; Figure 2C,D).

### 2.3. Rb1 Pretreatment Upregulated Antioxidant Genes in Cultured NPCs

**Figure 3:**
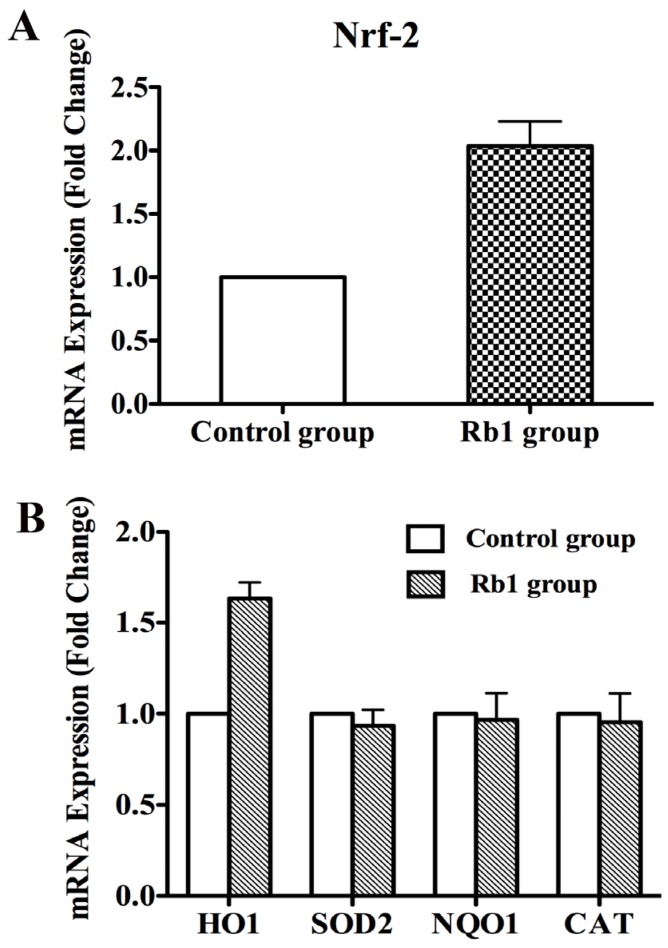
The mRNA expression levels of Nrf2, HO-1, SOD2, NQO1 and CAT in NPCs after pretreatment with Rb1. (**A**) Quantitative real-time PCR demonstrated that the expression level of Nrf2 increased by 2 fold in NPCs after treatment with 10 µM ginsenoside Rb1 for 24 h; (**B**) Quantitative real-time PCR to measure HO-1, SOD2, NQO1 and CAT levels in NPCs after pretreatment with 10 µM ginsenoside Rb1 for 24 h followed by incubation with 300 µM *t*-BHP for 2.5 h. The expression level of HO-1 was significantly elevated compared with the control group, whereas SOD2, NQO1 and CAT mRNA expression did not change after the same treatment.

We next sought to explore the underlying mechanism of Rb1-induced anti-oxidative cytoprotection. We first investigated whether Nrf2, the principal transcription factor that regulates the basal and inducible expression of a battery of antioxidant genes, was up-regulated after pretreatment with Rb1. Real-time RT-PCR assays showed that Rb1 pretreatment induced a 2-fold increase in the transcript level of Nrf2 when compared with the control group (Figure 3A). Furthermore, Rb1 pretreatment induced a 1.5-fold increase in the expression level of the downstream gene and phase II drug metabolizing enzyme HO-1 compared with the control. However, Rb1 made no contributions to activation of other downstream genes including SOD2, NQO1 and CAT (Figure 3B).

### 2.4. Pretreatment of Rb1 Showed No Effects on Promoting Proliferation and Neuronal Differentiation of NPCs

**Figure 4:**
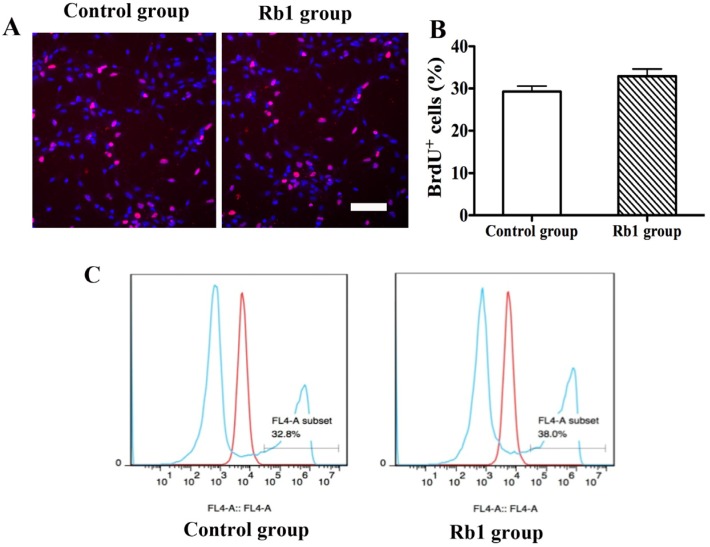
Effects of Rb1 on NPCs proliferation. (**A**) Representative photomicrographs of BrdU staining; (**B**) There was no difference in the percentage of BrdU-positive cells between the Rb1 group and control group. Eight representative fields per well and three wells per experiment were counted. Data were presented as means ± SEM from six experiments; (**C**) Flow cytometry analysis demonstrated that there was no difference in the percentage of proliferative cells between the Rb1 group and control group. Scale bar: 40 µm.

**Figure 5:**
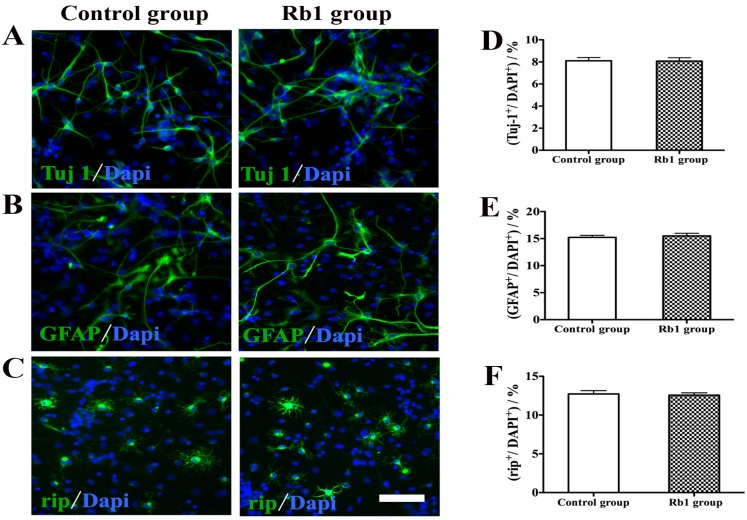
Effect of Rb1 on NPCs differentiation. (**A**–**C**) Representative photomicrographs of Tuj1 staining (**A**); GFAP staining (**B**); and Rip staining (**C**); (**D**–**F**) Rb1 pretreatment did not promote neuronal differentiation and alter glial generation by NPCs in culture and the percentages of Tuj-positive cells (**D**); GFAP-positive cells (**E**); and Rip-positive cells (**F**) did not differ between the Rb1 group and control group. Eight representative fields per well and three wells per experiment were counted. Data were presented as means±SEM from six experiments. Scale bar: 75 µm.

We also investigated whether pretreatment with Rb1 could enhance the proliferation and neuronal differentiation of NPCs. NPCs were treated with 10 µM Rb1 for 3 days. For proliferation studies, BrdU (10 µM) was added to the cultures 1 h before fixation of the cells. BrdU staining showed that there was no difference in the perecentage of BrdU-positive cells between the Rb1 group and control group (Figure 4A,B). Flow cytometry analysis using Click-iT EdU Flow Cytometry Assay Kits further demonstrated that there was no difference in the percentage of proliferative cells between the Rb1 group and control group (38.0% ± 0.16% *vs.* 32.8% ± 0.23%, *p >* 0.05; Figure 4C). For differentiation studies, pretreated NPCs were induced to differeniate in differentiation medium for 5 days. Rb1 pretreatment did not promote neuronal differentiation or alter glial generation by NPCs in culture (Figure 5). The percentages of βIII-tublin-positive cells, GFAP-positive cells, and Rip-positive cells did not differ between the Rb1 group and control group (*p >* 5; Figure 5A–F).

It is reported that ginsenosides have various beneficial effects in treatment of neurological disorders including Parkinson’s disease, Alzheimer’s disease, cerebral ischemia, depression [13]. In the present study, we showed that ginsenoside Rb1 exhibited potent neuroprotective effects on NPCs against oxidative injury and pretreatment with Rb1 activated Nrf2 pathway and led to an elevated expression of HO-1.

Ginsenoside Rb1 is one of the representative components of the ginsenosides. A number of preclinical and clinical studies have demonstrated that Rb1 possesses promising therapeutic potential in the treatment of oxidative injury [14,15,16]. Oxidative stress is one of the major causes of cell and tissue injury in a number of neurological disorders [17,18]. An interesting finding of the present study is that pretreatment with Rb1 significantly elevates Nrf2 expression in NPCs. Nrf2 pathway has been demonstrated to be an important endogenous anti-oxdative signal pathway in recent studies [19,20,21,22]. Our study demonstrated that its downstream antioxidant-responsive gene HO-1 was upregulated in Rb1-pretreated NPCs, indicating that the underlying mechanisms of the neuroprotective effects of Rb1 manifested the activation of the Nrf2/HO-1 pathway. Some studies have reported that Nrf2 activation leads to an elevated expression of other Nrf2-responsive genes including SOD2, NQO1 and CAT [23,24,25]. However, their expression levels did not change in Rb1-pretreated NPCs. The exact mechanisms underlying this expression profile remain unknown. It is of substantial value to explore the underlying mechanisms in the future studies. Heme oxygenase 1 is a 32 kDa enzyme encoded by the HO-1 gene. HO-1 has been reported to be the rate-limiting enzyme in heme catabolism. It has been found to be up-regulated during states of oxidative and cellular stress. It acts as a strong antioxidant with anti-inflammatory, anti-apoptotic, and immunomodulatory effects [26]. Therefore, HO-1 can protect cells against various injuries such as necrotizing enterocolitis and ischemic-reperfusion injury [27].

**Figure 6:**
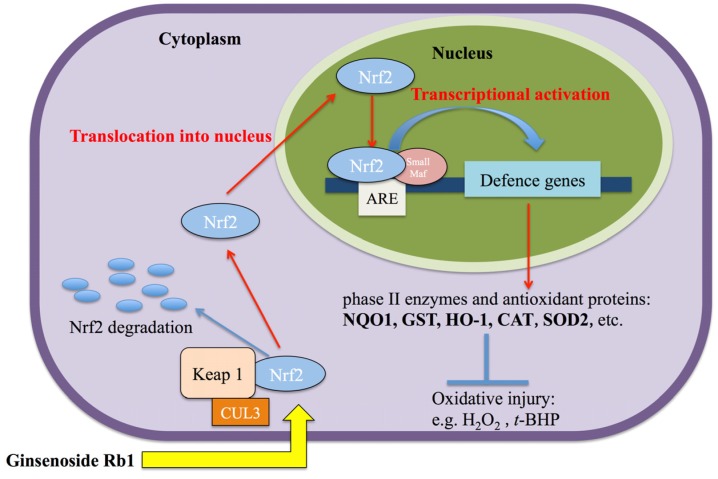
Proposed mechanisms underlying the cellular responses to t-BHP after pre-treatment with Rb1 via Nrf2/HO-1 signaling pathway.

How the Nrf2/HO-1 signaling pathway responds to oxidative stress on NPCs by pretreatment of Rb1 is shown in Figure 6.

Under normal conditions, Nrf2 is tightly bound to Keap1 and located in the cytoplasm where Nrf2 is ubiquitinated and subsequently degradated. Once stimulated, Nrf2 is not ubiquitinated and translocates into the nucleus. In the nucleus, it combines (a heterodimer) with a small Maf protein and binds to the Antioxidant Response Element (ARE) in the upstream promoter region of many antioxidative genes, and initiates their transcription. Our findings indicate that Rb1 activates the expression of Nrf2, and subsequently allows the nuclear translocation of Nrf2, which induces the expression of HO-1 and produces neuroprotective effects against oxidative injury.

## 3. Exprerimental

### 3.1. Materials

DMEM NUTRIENT MIX F12, fetal bovine serum(FBS), 0.05% (*w/v*) trypsin/1 mM EDTA, phosphate-buffered saline (PBS) powder, Goat serum, N2 supplement, B27 supplement were purchased from Gibco (Life Technologies Inc., Grand Island, NY, USA). Poly-l-lysine (PLL), laminin, 4',6-diamidino-2-phenylindole dihydrochloride (DAPI), bromodeoxyuridine (BrdU), bovine serum albumin (BSA), *tert*-butylhydroperoxide (*t*-BHP), paraformaldehyde, mouse anti-BrdU, rabbit anti-GFAP and mouse anti-β-tubulin III (Tuj1) were supplied by Sigma-Aldrich (St. Louis, MO, USA). Goat anti-mouse 488 antibody, goat anti-rabbit 568 antibody, Click-iT EdU Flow Cytometry Assay Kits, Qubit^®^ RNA BR Assay Kit were purchased from Invitrogen (Life Technologies Inc., Grand Island, NY, USA). Cytotoxicity Detection Kit (LDH) and In Situ Cell Death Detection Kit Fluorescein were obtained from Roche (Basel, Switzerland). RNeasy**^®^** Mini Kit was purchased from Qiagen (Düsseldorf, Germany). Primers, PrimeScript™ RT Master Mix (Perfect Real Time) Kit, and SYBR^®^ Premix Ex Taq™ II (Tli RNase H Plus) were supplied by Takara (Dalian, China). MicroAmp^®^ Optical 96-Well Reaction Plates with barcodes were purchased from Applied Biosystems (Life Technologies Inc., Grand Island, NY, USA). Mouse anti-Nestin was purchased from Millipore (Billerica, MA, USA). EGF and bFGF were purchased from Peprotech (Rocky Hill, NJ, USA). Mouse anti-Rip was a generous gift from X.M. Xu, University of Louisville, KY, USA. Ginsenoside-Rb1 is a reference compound (purity > 98%) was obtained from Chengdu Must Bio-Technology Co., Ltd. (Chengdu, China). A stock solution of Rb1 (5 mM) was prepared in sterile saline. All other chemicals and reagents were of analytical grade.

### 3.2. Cell Isolation and Culture

Under sterile conditions, cerebral cortex from E14.5 Sprague-Dawley (SD) rats were dissected out and prepared for NPCs culture following procedures described previously [28,29]. Briefly, the cortex was separated from surrounding tissues. After peeling off the meninges, the cortex was transferred into a 15 mL centrifuge tube containing culture medium (described below) and dissociated to a single-cell suspension by gentle mechanical trituration through a fire polished Pasteur pipette. The dissociated cells were filtered through a cell strainer (BD Falcon, NJ, USA) and then cultured in T25 flask in suspension. The culture medium consisted of DMEM-F12, BSA (1 mg/mL), B27 (20 µL/mL), N2 (10 µL/mL), EGF (20 ng/mL), and bFGF (20 ng/mL). Cells were maintained in an incubator with a humidified atmosphere containing 5% CO_2_ at 37 °C. The medium was changed every 2 days. After 5–6 days, cells grew in neurospheres with the diameter of apporimately 150 µm. Cells in the neurospheres were passaged at the ratio of 1:6. These subcultured cells were designated as “first passage” (P1). The third passage (P3) cells were used for all the following experiments.

### 3.3. Establishment of t-BHP Induced Oxidative Injury Model

The P3 NPCs were dissociated into single cells and seeded in 96-well plates coated with poly-l-lysine (13.3 µg/mL) and laminin (20 µg/mL) at a density of 1 × 10^4^ cells per well. The cultures were grown at 37 °C in a humidified CO_2_ incubator for 12 h and then were treated with 50, 100, 200 and 300 μM *t*-BHP for 2.5 h. Cytotoxicity of *t*-BHP to the whole cells culture was determined using LDH cytotoxicity assay. Approximately 35%–45% toxicity rate induced by t-BHP at specific concentrations was considered to be an optimal *t*-BHP oxidative stress model.

### 3.4. Lactate Dehydrogenase (LDH) Assay

Cellular injury of NPCs was determined by measuring the activity of LDH released into the incubation medium. Released LDH activity was determined by the cytotoxicity detection kit. The absorbance of the samples at 490 nm was measured according to the filter available by SpectraMax M5 Multi-Mode Microplate Reader (Molecular Devices, Sunnyvale, CA, USA) at 490 nm. Each experiment was independently repeated three times.

### 3.5. Treatment of NPCs with Rb1 in a t-BHP Oxidative Injury Model

The P3 NPCs were seeded on 96-well plates at a density of 1 × 10^4^ cells per well. The cultures were grown at 37 °C in a humidified CO_2_ incubator for 12 h. Rb1 was added to the cell culture medium (final concentration: 0, 0.1, 1, 10, and 100 µM) for 24 h, followed by drug washout before experiments. The cells were followed by treatment with 300 μM *t*-BHP for another 2.5 h, and the cell viability was measured by the LDH assay and further confirmed with TUNEL staining.

### 3.6. TUNEL Staining

A TUNEL assay was used to assess the apoptotic cells by *in situ* cell death detection kit according to the manufacturer’s instructions. Firstly, fixed cell samples were incubated in the TUNEL reaction medium for 1 h at 37 °C in the dark. After the reaction was completed, the cells were washed by PBS, transferred into a 2 μg/mL DAPI solution, and mounted on slides. The number of apoptotic nuclei and total number of nuclei were determined under a fluorescence microscope.

### 3.7. Immunocytochemistry

Dissociated cells in single cell suspension were plated on cover slips coated with PLL/laminin (1:1 ratio) at a density of 10^4^/cm^2^ in a 24-well plate. For proliferation studies, BrdU (10 µM) was added to cultures 1 h before fixation of the cells. For differentiation studies, growth factors were removed from the culture medium and 1% FBS was added. The cultures were allowed to differentiate for up to 5 days. The cells on coverslips were fixed in 4% paraformaldehyde dissolved in 0.1 M phosphate buffer (PB) for 20 min. After several washes with 0.01 M PBS, cells were processed for immunocytochemistry. The following primary antibodies were used to stain the cells: monoclonal anti-βIII-tublin (1:500) for neurons; polyclonal anti-glial fibrillary acidic protein (GFAP) antibody (1:1,000), and monoclonal anti-Rip antibody (1:50) for astrocytes and oliogodendrocytes, respectively; and monoclonal anti-BrdU (1:100) for BrdU-incorporating cells. The cultures were incubated with the primary antibodies in PBS plus 1% BSA, 2% normal goat serum and 0.3% Triton X-100 for 2 h at room temperature. Primary antibodies were visualized with species-specific secondary antibody conjugated to the fluorescent labels Alexa 568 or 488 (1:400). Cells were mounted in anti-fade medium containing DAPI to counterstain nuclei. The fluorescence images were captured using a fluorescence microscope.

### 3.8. Quantitative Real-Time PCR

Cells were pretreated by 10 µM Rb1 for 24 h, then the total RNA of 10 µM Rb1 group and control group was isolated by using the RNeasy^®^ Mini Kit [30]. RNA concentrations were determined using a NanoDrop 2000 (Thermo Scientific, Waltham, MA, USA) with Qubit^®^ RNA BR Assay Kit. Reverse transcription was performed by using the PrimeScript™ RT Master Mix Kit according to the manufacturer’s protocol.

Amplifications were performed in duplicate in 20 ul reaction volumes containing 1×SYBR^®^ Premix Ex Taq™ II (Tli RNase H Plus), 0.2 uM of each primer, and 2 ul target DNA to quantitatively detect the geen expression of Nrf2, HO-1, SOD2,NQO1 and CAT [31]. All primer sequences were listed in Table 1. Reaction conditions were an initial denaturation at 95 °C for 30 s, followed by 40 cycles of 95 °C for 5 s, 60 °C for 34 s, followed by melt curve analysis by 7500 Real-Time PCR System (Applied Biosystems).

**Table 1:** PCR primers used in the gene expression analysis.

*Gene name*	*Forward (5') primer*	*Reverse (3') primer*	*Association No.*
Beta actin	5'-GTCGTACCACTGGCATTCTG-3'	5'-CTCTCAGCTGTGGTGGTGAA-3'	NM_031144
NRF-2	5'-gCAACTCCAgAAggAACAgg-3'	5'-CAgTgAggggATCgATgAgT-3'	NM-031789.1
HO-1	5'-TgCTCgCATgAACACTCTg-3'	5'-TCCTCTgTCAgCAgTgCCT	NM_012580.2
SOD2	5'-ggCCAAgggAgATgTTACAA-3'	5'-gCTTgATAgCCTCCAgCAAC-3'	NM_001274771
NQO1	5'-gCCCggATATTgTAgCTgAA-3'	5'-gTggTgATggAAAgCAAggT-3'	NM_017000.3
CAT	5'-TTATggCCTCCgAgATCTTTTC-3'	5'-ACCTTggTCAggTCAAATggAT-3'	NM_012520

### 3.9. Statistical Analysis

Statistical differences between two groups were determined by two-tailed Student’s t test. Multiple group comparisons were made by one-way ANOVA and Tukey post hoc test. Data were presented as mean ± SEM. Significance levels were set to 0.05 for all comparisons.

## 4. Conclusions

Our study demostrated that Rb1 shows potent neuroprotective effects against oxidative injury via activation of the Nrf2/HO-1 signaling pathway. These results support further exploration of Rb1 as an effective antioxidant for treating neurological diseases.
